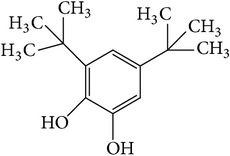# *In Vitro* Anticancer Activity of a Nonpolar Fraction from *Gynostemma pentaphyllum* (Thunb.) Makino

**DOI:** 10.1155/2016/6308649

**Published:** 2016-02-29

**Authors:** Yantao Li, Jiajun Huang, Wanjun Lin, Zhongwen Yuan, Senling Feng, Ying Xie, Wenzhe Ma

**Affiliations:** State Key Laboratory of Quality Research in Chinese Medicine, Macau University of Science and Technology, Macau

## Abstract

*Gynostemma pentaphyllum* (Thunb.) Makino (GpM) has been widely used in traditional Chinese medicine (TCM) for the treatment of various diseases including cancer. Most previous studies have focused primarily on polar fractions of GpM for anticancer activities. In this study, a nonpolar fraction EA1.3A from GpM showed potent growth inhibitory activities against four cancer cell lines with IC_50_ ranging from 31.62 *μ*g/mL to 38.02 *μ*g/mL. Furthermore, EA1.3A also inhibited the growth of breast cancer cell MDA-MB-453 time-dependently, as well as its colony formation ability. EA1.3A induced apoptosis on MDA-MB-453 cells both dose-dependently and time-dependently as analyzed by flow cytometry and verified by western blotting analysis of apoptosis marker cleaved nuclear poly(ADP-ribose) polymerase (cPARP). Additionally, EA1.3A induced cell cycle arrest in G0/G1 phase. Chemical components analysis of EA1.3A by GC-MS revealed that this nonpolar fraction from GpM contains 10 compounds including four alkaloids, three organic esters, two terpenes, and one catechol substance, and all these compounds have not been reported in GpM. In summary, the nonpolar fraction EA1.3A from GpM inhibited cancer cell growth through induction of apoptosis and regulation of cell cycle progression. Our study shed light on new chemical bases for the anticancer activities of GpM and feasibilities to develop new anticancer agents from this widely used medicinal plant.

## 1. Introduction

*Gynostemma pentaphyllum* (Thunb.) Makino (GpM), also called* Jiaogulan*, is known as a folk medicine in the Chinese population for centuries for treating various ailments such as hematuria, edema in the pharynx and neck, and trauma. Modern pharmacology has demonstrated that GpM exhibits a variety of pharmacological activities including anti-inflammatory [1], regulation of lipid metabolism [2], antioxidative [3], neuroprotective [4], and anxiolytic effects [5].

The anticancer activity of GpM was first brought to the forefront in the 1970s. A nationwide population census revealed that, in some small villages in the southwest provinces of China, regularly GpM tea consuming was associated with longevity and cancer-free survival [6]. A host of studies were then carried out to isolate bioactive compounds [7, 8] and to test anticancer activities on cells [9, 10], animals [11, 12], and patients [13, 14]. Multiple mechanisms have been proposed for extracts/compounds from GpM with anticancer activities, including cell cycle arrest [15], apoptosis induction [16], inhibition of invasion and metastasis [17], glycolysis inhibition [18], and immune modulation [19].

More than 210 compounds have been identified from GpM so far. Based on their chemical structures, these compounds could be grouped into saponin, sterol, flavonoid, and polysaccharide. Among these groups, saponin (named gypenoside, Gyp) is the largest family containing about 170 members and is reported as the major active ingredients of GpM [8, 20–22]. Other components of GpM, like flavonoids [23, 24] and polysaccharides [25, 26], also exhibit various biological activities, while sterols are rarely reported.

In most phytochemical studies of GpM, the water soluble extracts [26–29], the ethanol or methanol extracts [30–32], or fractions eluted with polar solvent [9, 23, 32] were focused on. Few studies regarding the nonpolar fractions of GpM have been carried out. Piao et al. extracted GpM with ethyl acetate, but, in the following steps of elution and isolation, polar solvents like ethanol, methanol, and water were applied, which indicated that the final compounds or ingredients were still polar [22].

To date, most of the reported anticancer components from GpM are polar fractions, like Gyps, flavonoids, and polysaccharides. In this work, we focused on the nonpolar fraction of GpM. After extracting with ethyl acetate, nonpolar solvents like n-hexane and trichloromethane were used as the elution solvents at the chromatographic separation step. The obtained fraction (EA1.3A) was thus nonpolar and showed potent anticancer effects* in vitro*. And chemical characterization of EA1.3A by GC-MS analysis found that compounds have not been reported in GpM previously which may contribute to its anticancer activities.

## 2. Materials and Methods

### 2.1. Plant Material

*Gynostemma pentaphyllum* (Thunb.) Makino was purchased from the local drug store in Zhuhai, Guangdong, China.

### 2.2. Chemicals

RPMI 1640 medium, McCoy's 5A (Modified) medium, fetal bovine serum (FBS), penicillin-streptomycin (10,000 U/mL), and TrypLE*™* Express enzyme were purchased from GIBCO. Sulforhodamine B (SRB), Trizma® base, and monoclonal anti-*β*-actin antibody were purchased from Sigma. Annexin V: FITC Apoptosis Detection Kit I was purchased from BD Biosciences and cell cycle detect kit was purchased from Nanjing Key GEN BioTECH. Cleaved nuclear poly(ADP-ribose) polymerase (cPARP) mouse monoclonal antibody was purchased from Cell Signaling.

### 2.3. Extraction and Fractionation

The dry aerial parts of* Gynostemma pentaphyllum* (Thunb.) Makino (3,000 g) were pulverized and exhaustively extracted twice using 75% ethanol (15,000 mL) at room temperature for two days. The supernatants were combined, filtered, and evaporated under vacuum by means of a rotary evaporator to obtain a dried extract (119.2 g). The ethanol extract was then resuspended in 1,000 mL water and extracted successively using 1,000 mL of solvents with increasing polarity: hexane, ethyl acetate, and n-butanol. The ethyl acetate extract (18.9 g) was subjected to silica gel column and eluted with hexane : ethyl acetate (6 : 4) followed by chromatographic separation using Sephadex LH-20 and eluted with CHCl3 : MeOH (60 : 40). The final extract was named EA1.3A (32.5 mg).

### 2.4. Cell Lines and Culture

All cell lines were purchased from Cell Bank of Chinese Academy of Science, Shanghai, China. Human breast adenocarcinoma cell lines MDA-MB-453 and MCF7 and human prostatic adenocarcinoma cell line LNCaP were cultured in RPMI-1640 medium. Human colorectal carcinoma cell line HCT116 was cultured in McCoy's 5A medium. Normal human lung fibroblast cell line CCD-19Lu was cultured in MEM medium. All media were supplemented with 10% FBS, 100 units/mL penicillin G, and 100 *μ*g/mL streptomycin. All cell lines were grown at 37°C under 5% CO_2_ in a humidified atmosphere.

### 2.5. *In Vitro* Cell Proliferation Assay (SRB Assay)

The antiproliferative effects of fraction EA1.3A on cancer cell lines were assessed by sulforhodamine B (SRB) colorimetric assay as previously described [33]. Briefly, cells were seeded in 96-well plates in a volume of 100 *μ*L/well at densities of 5,000 cells per well. After overnight incubation at 37°C in a humidified incubator with 5% CO_2_, 100 *μ*L medium containing EA1.3A (2x indicated concentrations) was added. After treatment for indicated times, attached cells were fixed with 50 *μ*L cold 50% (w/v) trichloroacetic acid (TCA) for 1 hour at 4°C, washed 5 times with slow-running tap water, and stained with 100 *μ*L 0.4% (w/v) SRB. The absorbency at 515 nm was measured using SpectraMax 190 microplate reader (Molecular Devices) after solubilizing the protein-bound dye with 200 *μ*L 10 mM Tris base solution (pH 10.5). The relative cell growth rate was determined using the following equation: Relative Growth (%) = OD (treated)/OD (control). The IC_50_ value was defined as the concentration required for a 50% reduction in cell growth.

### 2.6. Colony Formation Assay

MDA-MB-453 cells were plated at 4,000 cells/well on six-well plates and cultured with indicated concentrations of EA1.3A for 12 days. Plates were stained with 0.2% (w/v) crystal violet in buffered formalin for 20 minutes, and colonies were then imaged and quantified.

### 2.7. Analysis of Apoptosis by Flow Cytometry

Cellular apoptosis was analyzed with BD Annexin V: Fitc Apoptosis Detection Kit I (BD Bioscience) by flow cytometry. MDA-MB-453 cells were treated with EA1.3A at indicated concentrations for 72 hours. Then cells were harvested, washed twice with PBS, and resuspended in binding buffer at a concentration of 1,000,000 cells/mL. Cells (100 *μ*L) were transferred to 1.5 mL conical tube and stained with FITC Annexin V (5 *μ*L) and propidium iodide (5 *μ*L) at room temperature for 15 minutes in the dark. Cells were filtered and analyzed by flow cytometry (BD Bioscience) within 1 hour. Total apoptotic cells (FITC Annexin V positive) were counted.

### 2.8. Analysis of Cell Cycle by Flow Cytometry

MDA-MB-453 cells were treated with EA1.3A at indicated concentrations for 72 hours. Then cells were harvested, washed twice with PBS, and fixed in 70% ethanol at −20°C for 2 hours. Cells were stained for total DNA content in propidium iodide (PI) staining solution (20 *μ*g/mL PI, 200 *μ*g/mL DNase-free RNase A, and 0.1% triton X-100 in PBS) for 30 minutes at room temperature in the dark. Cell cycle distribution was analyzed by flow cytometry (BD Bioscience). The percentage of the total cell population in the four different phases (Sub-G0/G1, G0/G1, S, G2/M) of cell cycle was determined using FlowJo software.

### 2.9. Western Blotting

MDA-MB-453 cells were treated with EA1.3A at indicated concentrations for 72 hours. Protein samples were prepared by scratching cells in RIPA buffer containing protease inhibitor cocktail (Roche) and diluted in SDS-PAGE protein sample buffer. Samples were heated for 5 minutes at 95°C and equal amount of proteins was subjected to electrophoresis on SDS-polyacrylamide gels. The proteins were then transferred to PVDF membranes (Millipore) and incubated with primary antibodies overnight at 4°C. The membranes were then washed with TBST and incubated with the appropriate horseradish peroxidase-conjugated secondary antibodies at room temperature. Proteins were visualized with SuperSignal West Dura Extended Duration Substrate (Thermo Scientific).

### 2.10. GC-MS Analysis

A method based on Karthikeyan et al. was employed to character structures of components from fraction EA1.3A [34]. In brief, EA1.3A was analyzed on an Agilent 6890 gas chromatograph coupled with an Agilent 5973 mass selective detector. Compounds were separated on a DB-1ms capillary column (30 m × 0.250 mm i.d., 0.25 *μ*m film thickness) (J&W Scientific, Folsom, CA, USA). Carrier gas was helium at 1.0 mL/min. One microliter of the sample was injected into GC-MS using split mode (10 : 1). The injection port temperature was maintained at 250°C. The column oven temperature was held at 80°C for 2 min, then programmed at 10°C/min to 250°C and held for 0 min, and then programmed at 5°C/min to 280°C and held for 9 min. Electron impact spectra in positive ionization mode were acquired between *m*/*z* 40 and 450. The spectrum obtained after GC-MS analysis was interpreted and compared with the National Institute Standard and Technology (NIST) compounds library [35].

## 3. Results

### 3.1. Antiproliferative Activity of EA1.3A

**Figure 1:**
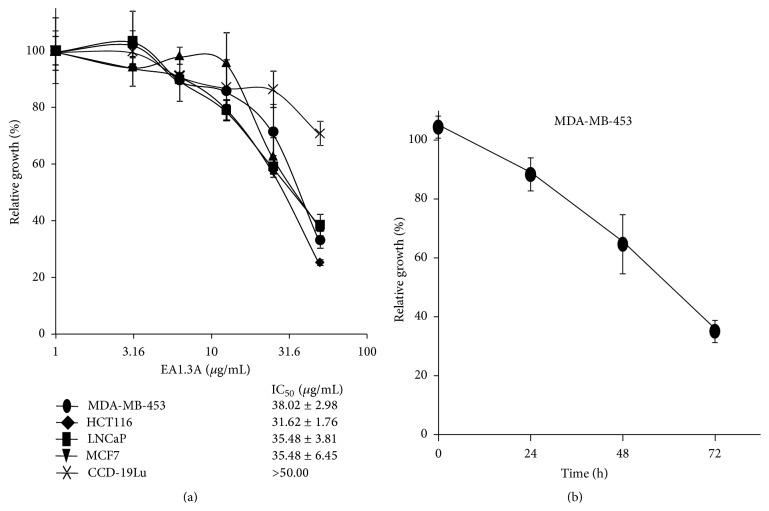
EA1.3A inhibits proliferation of cancer cell lines. (a) Dose effect of EA1.3A treatment (72 hours) on the proliferation of two breast cancer cell lines (MCF7 and MDA-MB-453), one colon cancer cell line (HCT116), one prostate cancer cell line (LNCaP), and one normal human lung fibroblast cell line (CCD-19Lu). The cell number at each EA1.3A concentration is represented as a percentage of control. Average values are from three independent experiments performed in duplicate (*n* = 3). (b) Time course of EA1.3A treatment (50 *μ*g/mL) on the proliferation of breast cancer cell line MDA-MB-453. The cell number at each time point is represented as a percentage of control. Average values are from three independent experiments performed in duplicate (*n* = 3). Data are shown as mean ± SD.

In an attempt to search for components with anticancer activity from GpM, we found the nonpolar fraction EA1.3A exerted greater growth inhibitory activity than other fractions on several cancer lines (data not shown). So we focused on this fraction and systematically evaluated its anticancer activity* in vitro*. Two breast cancer cell lines (MCF7 and MDA-MB-453), one colon cancer cell line (HCT116), one prostate cancer cell line (LNCaP), and one normal human lung fibroblast cell line (CCD-19Lu) were treated with a serial of EA1.3A concentrations from 2 *μ*g/mL to 50 *μ*g/mL for 72 hours and the cytotoxic activity was evaluated by SRB assay. It was shown that EA1.3A inhibited the growth of all four cancer cell lines dose-dependently, with IC_50_ values ranging from 31.62 *μ*g/mL to 38.02 *μ*g/mL (Figure 1(a)), while the normal human lung fibroblast cell line CCD-19Lu was relative resistant to EA1.3A treatment (IC_50_ is greater than 50 *μ*g/mL, the highest concentration tested in the study), which implies the specific anticancer activities of this nonpolar fraction from GpM (Figure 1(a)). To further demonstrate the anticancer activity of EA1.3A, we chose breast cancer cell line MDA-MB-453, the relative less sensitive cell line, for the rest of our studies.

**Figure 2:**
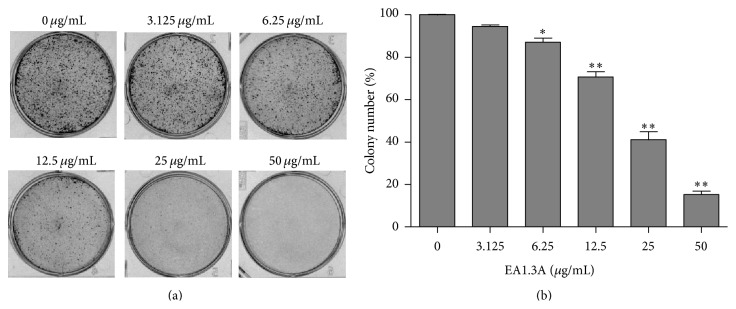
Colony formation of MDA-MB-453 cells with EA1.3A treatment. (a) Representative colony formation assay plates of MDA-MB-453 cells treated with EA1.3A at indicated concentrations for 12 days. (b) Quantification of colony number (*n* = 3). Data are shown as mean ± SD. *P* values determined by Student's *t*-test. ^*∗*^ *P* < 0.001; ^*∗∗*^ *P* < 0.0001.

Consistent with the dose effect of EA1.3A on cancer cell growth (Figure 1(a)), when treated with 50 *μ*g/mL of EA1.3A, the growth of MDA-MB-453 cells was also inhibited in a time-dependent manner. After treatment for 24 hours, the relative growth rate of MDA-MB-453 cells was 88.34% and declined to 35.12% after 72 hours (Figure 1(b)). So EA1.3A inhibited the growth of breast cancer cell MDA-MB-453 both dose- and time-dependently. This was further confirmed by colony formation assay (Figure 2). Colony formation ability of MDA-MB-453 cell was significantly decreased after treatment with EA1.3A at 6.25 *μ*g/mL and almost abolished at 50 *μ*g/mL.

### 3.2. Analysis of Apoptosis

**Figure 3:**
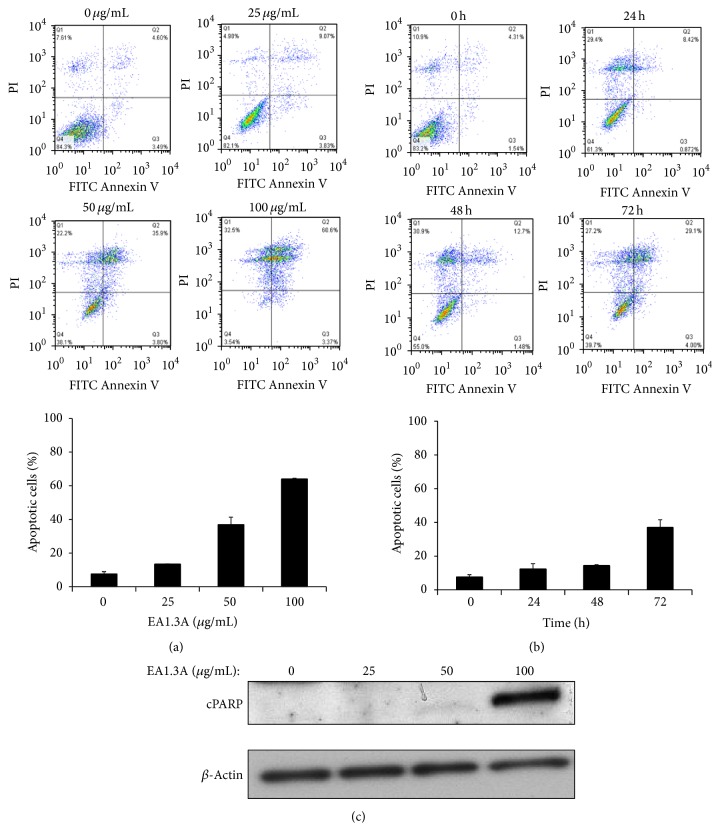
EA1.3A induces apoptosis in MDA-MB-453 cells. After treatment with EA1.3A, breast cancer cells MDA-MB-453 were stained with FITC Annexin V/PI and apoptosis was quantified by flow cytometry. (a) Dose effect of EA1.3A treatment (72 hours) on apoptosis. Average values are from three independent experiments (*n* = 3). (b) Time course of EA1.3A treatment (50 *μ*g/mL) on apoptosis. Average values are from three independent experiments (*n* = 3). (c) Western blotting analysis of apoptosis marker cleaved nuclear poly(ADP-ribose) polymerase (cPARP). Data are shown as mean ± SD.

The nonpolar fraction EA1.3A of GpM showed potent antiproliferative activity against cancer cells as described above. And we observed typical morphological characteristics of apoptosis of cells treated with EA1.3A, like chromatin condensation and nuclear fragmentation (data not shown). To further elucidate whether the growth inhibitory activity of EA1.3A was induced by activation of apoptosis, we analyzed apoptosis of MDA-MB-453 cells after treatment with EA1.3A by flow cytometry with annexin V and propidium iodide double staining. As shown in Figure 3(a), EA1.3A induced apoptosis of MDA-MB-453 cells in a dose-dependent manner. The percentages of apoptotic cells (Annexin V positive) with treatment of EA1.3A for 72 hours were 12.9% at 25 *μ*g/mL, 39.7% at 50 *μ*g/mL, and 64.0% at 100 *μ*g/mL, respectively. EA1.3A induced apoptosis also in a time-dependent manner. When MDA-MB-453 cells were treated with EA1.3A at 50 *μ*g/mL for 24 hours, 9.3% cells underwent apoptosis. The number increased to 14.2% and 33.1% for 48 hours and 72 hours, respectively (Figure 3(b)). So, EA1.3A induced apoptosis of MDA-MB-453 cells in both dose- and time-dependent manner. The apoptosis inducing ability of EA1.3A was further confirmed by the detection of the cleaved nuclear poly(ADP-ribose) polymerase (cPARP), a marker of apoptosis, in MDA-MB-453 cells after treatment with EA1.3A by western blotting (Figure 3(c)).

### 3.3. Analysis of Cell Cycle

**Figure 4:**
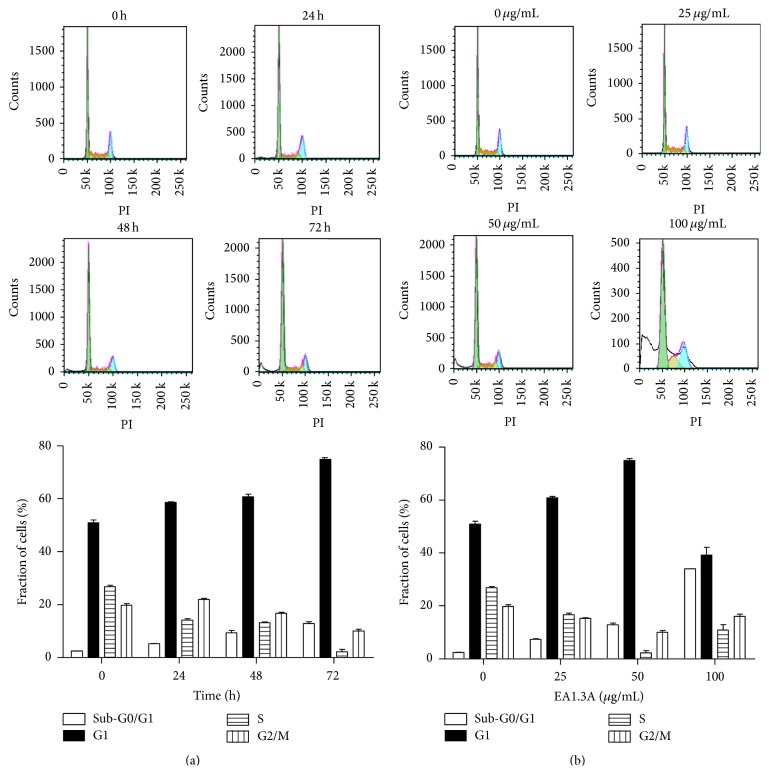
EA1.3A induces cell cycle arrest in MDA-MB-453 cells. After treatment with EA1.3A, cell cycle distributions of breast cancer cells MDA-MB-453 were analyzed by flow cytometry and percentages of the total cell population in the four different phases of cell cycle (Sub-G0/G1, G0/G1, S, and G2/M) were determined using FlowJo software. (a) Time course of EA1.3A treatment (50 *μ*g/mL) on cell cycle progression. Average values are from three independent experiments (*n* = 3). (b) Dose effect of EA1.3A treatment (72 hours) on cell cycle progression. Average values are from three independent experiments (*n* = 3). Data are shown as mean ± SD.

Cell cycle arrest can also cause reduced proliferation, so we next analyzed cell cycle distribution of MDA-MB-453 cells treated with EA1.3A. As shown in Figure 4(a), the percentages of cells in G0/G1 phase were slightly increased with time after EA1.3A treatment (58.65% at 24 hours, 60.74% at 48 hours, and 74.91% at 72 hours comparing with 50.94% of the control). The time-dependent increase of cells in G0/G1 phase was accompanied with decreasing number of cells in S phage (26.85% of the control, 14.14% at 24 hours, 13.14% at 48 hours, and 2.24% at 72 hours) and in G2/M phase (2.43% of the control, 5.23% at 24 hours, 9.38% at 48 hours, and 12.81% at 72 hours). So it indicates that EA1.3A can cause cells arrest in G0/G1 phase, which was further confirmed by analysis of cells treated with EA1.3A at various concentrations (Figure 4(b)). The percentages of MDA-MB-453 cells in G0/G1 phase were 50.64% of the control, 60.79% at 25 *μ*g/mL, and 74.91% at 50 *μ*g/mL, respectively, while at 100 *μ*g/mL, there was a remarkable drop of G0/G1 proportion, probably due to cells undergoing a massive apoptosis in the circumstances (Figure 3(a)). In short, EA1.3A induced cell cycle arrest in G0/G1 phase, which may also contribute to its antiproliferative activity. It is also notable that the proportion of cells in sub-G0/G1 phase was also increased in both time- (Figure 4(a)) and dose-dependent (Figure 4(b)) manner, which is consistent with apoptosis analysis of cells treated with EA1.3A as discussed above.

### 3.4. GC-MS Analysis of EA1.3A

**Figure 5:**
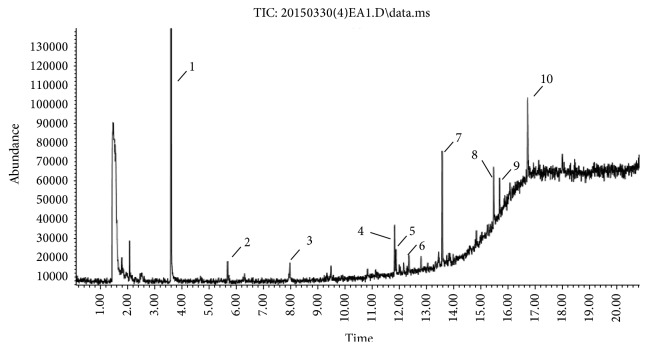
Gas chromatography of EA1.3A.

The nonpolar fraction EA1.3A from GpM, which is less studied, showed potent anticancer activities in this study. We next tried to delineate the chemical components of this fraction. Identification and chemical analysis of EA1.3A by GC-MS is displayed in Table 1. The chromatogram (Figure 5) revealed a total of ten peaks which were identified as propanoic acid, 2-methylpropyl ester (42.7%), *β*,*β*-carotene-3,3′-diol,5,8-epoxy-5,8-dihydro,(3S,3′R,5R,8S)- (1.2%), cyclopropanecarboxamide, 2-phenyl-N-[4-(phenylazo)phenyl]- (7.9%), heptyl phthalate (3.3%), 2,4-dimethylbenzo[h]quinolone (2.0%), 2-methyl-7-phenylindole (1.4), phytol (6.6%), 5-methyl-2-phenylindolizine (5.2%), phthalic acid, 2-cyclohexylethyl propyl (5.2%), and 3,5-Di-tert-butylcatechol (17.6%). Among these compounds, four (peaks number 3, 5, 6, and 8) are alkaloids, three (peaks number 1, 4, and 9) are organic esters, two (peaks number 2 and 7) are terpenes and one (peak number 10) which belong to catechol substance. To our knowledge, all these 10 compounds have not been reported in GpM, probably because the nonpolar fractions from GpM have been neglected by previous studies.

## 4. Discussion

To date, most reported components of GpM with anticancer activities are polar fractions/compounds, like Gyps, flavonoids, and polysaccharides. Here, for the first time, we showed that the nonpolar fraction EA1.3A from GpM also has potent anticancer activities through regulation of cell cycle progression and induction of apoptosis. EA1.3A even exhibited greater anticancer activities than other fractions (data not shown) with IC_50_ values ranging from 31.62 *μ*g/mL to 38.02 *μ*g/mL against 4 cancer cell lines tested in this study which are also comparable with or more potent than reported polar fractions from GpM. For example, the IC_50_ values of Gyps were of 39.3 *μ*g/mL on PC-3 cells [23], 112.39 *μ*g/mL on Eca-109 cells, and 61.68 *μ*g/mL on SW620 cells [36], the IC_50_ value of flavonoids was of 33.3 *μ*g/mL on PC-3 cells [23], and the IC_50_ value of polysaccharide was of 65.4 *μ*g/mL on B16 cells [26].

GC-MS analysis identified 10 compounds in EA1.3A and all of them have not been reported in GpM. It was not surprising to identify three organic esters, which are usually nonpolar, in fraction EA1.3A from GpM. The major component of EA1.3A was propanoic acid, 2-methylpropyl ester (isobutyl propionate), which is widely used in food and beverage industries as a rum flavor [37]. Although there is no anticancer activity reported for this compound, the parent acid propionate inhibits cancer cell proliferation through multiple mechanisms [38–41]. Alkaloids are widely distributed in plants characterized with basic nitrogen atoms and have a wide range of pharmacological activities [42]. Although few alkaloids have been isolated from GpM, in the present study, we identified four alkaloids in EA1.3A. One of them, 2-methyl-7-phenylindole, has been found present in a variety of plants [43–48], and extract containing 2-methyl-7-phenylindole showed cytotoxicity effects [44]. Two terpenes were identified in EA1.3A. One of them, *β*-carotene-3,3′-diol,5,8-epoxy-5,8-dihydro,(3S,3′R,5R,8S)- belongs to xanthophylls, one kind of carotenoids, which are widely presented in the leaves of most green plants. Xanthophylls are known for their antioxidant activities and have been found to reduce oxidative stress and prevent tumorigenesis [49, 50]. Phytol, a constituent of chlorophyll, is a precursor for the manufacture of synthetic forms of vitamin E [42] and vitamin K1 [51]. Extensive studies have proven anticancer activities of phytol [52–55]. The catechol substance, 3,5-di-tert-butylcatechol, was identified to be an effective inhibitor of the enzyme sarco/endoplasmic reticulum calcium ATPase (SERCA), a potential target for cancer chemotherapy, by virtual screens and confirmed by bioassays [56]. These compounds may contribute to the anticancer activities of nonpolar fraction EA1/3A from GpM.

## 5. Conclusions

In summary, the rarely studied nonpolar fraction EA1/3A from GpM showed potent anticancer activities via induction of cell cycle arrest and apoptosis. Our study shed light on new chemical bases for the anticancer activities of this widely used medicinal herb. Further studies to isolate and identify pure compounds from this nonpolar fraction from GpM and evaluate the anticancer activities as well as the mechanisms of action of these compounds are suggested for developing novel anticancer agents.

## Figures and Tables

**Table 1:** Compounds identified from EA1.3A fraction from GpM by GC-MS analysis.

Peak number	RT (min)	Compound	Peak area (%)	Molecular structure
1	3.59	Propanoic acid, 2-methylpropyl ester	42.7	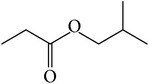

2	5.68	*β*,*β*-carotene-3,3′-diol,5,8-epoxy-5,8-dihydro,(3S,3′R,5R,8S)-	1.2	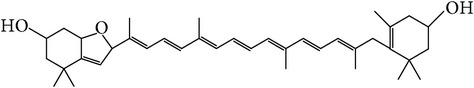

3	7.97	Cyclopropanecarboxamide,2-phenyl-N-[4-(phenylazo)phenyl]-	7.9	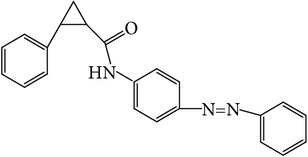

4	11.83	Heptyl phthalate	3.3	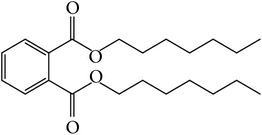

5	11.88	2,4-Dimethylbenzo[h]quinoline	2.0	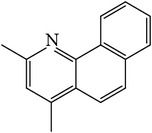

6	12.35	2-Methyl-7-phenylindole	1.4	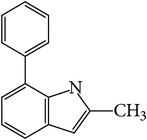

7	13.58	Phytol	6.6	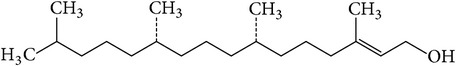

8	15.47	5-Methyl-2-phenylindolizine	5.2	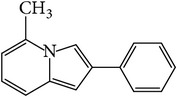

9	15.69	Phthalic acid, 2-cyclohexylethyl propyl	5.2	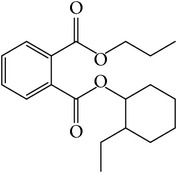

10	16.72	3,5-Di-tert-butylcatechol	17.6